# Imaging acquisition display performance: an evaluation and discussion of performance metrics and procedures

**DOI:** 10.1120/jacmp.v17i4.6220

**Published:** 2016-07-08

**Authors:** Michael S. Silosky, Rebecca M. Marsh, Ann L. Scherzinger

**Affiliations:** ^1^ Radiology, School of Medicine, University of Colorado Denver Aurora CO USA

**Keywords:** liquid crystal displays, medical image display, quality assurance, luminance, display uniformity

## Abstract

When The Joint Commission updated its Requirements for Diagnostic Imaging Services for hospitals and ambulatory care facilities on July 1, 2015, among the new requirements was an annual performance evaluation for acquisition workstation displays. The purpose of this work was to evaluate a large cohort of acquisition displays used in a clinical environment and compare the results with existing performance standards provided by the American College of Radiology (ACR) and the American Association of Physicists in Medicine (AAPM). Measurements of the minimum luminance, maximum luminance, and luminance uniformity, were performed on 42 acquisition displays across multiple imaging modalities. The mean values, standard deviations, and ranges were calculated for these metrics. Additionally, visual evaluations of contrast, spatial resolution, and distortion were performed using either the Society of Motion Pictures and Television Engineers test pattern or the TG‐18‐QC test pattern. Finally, an evaluation of local nonuniformities was performed using either a uniform white display or the TG‐18‐UN80 test pattern. Displays tested were flat panel, liquid crystal displays that ranged from less than 1 to up to 10 years of use and had been built by a wide variety of manufacturers. The mean values for $L_{\min}$ and $L_{\max}$ for the displays tested were $0.28\pm 0.13\;\text{cd}/m^{2}$ and $135.07\pm 33.35\;\text{cd}/m^{2}$, respectively. The mean maximum luminance deviation for both ultrasound and non‐ultrasound displays was 12.61%±4.85% and 14.47%±5.36%, respectively. Visual evaluation of display performance varied depending on several factors including brightness and contrast settings and the test pattern used for image quality assessment. This work provides a snapshot of the performance of 42 acquisition displays across several imaging modalities in clinical use at a large medical center. Comparison with existing performance standards reveals that changes in display technology and the move from cathode ray tube displays to flat panel displays may have rendered some of the tests inappropriate for modern use.

PACS number(s): 87.57.‐s, 87.57.C‐

## I. INTRODUCTION

Recently, The Joint Commission (TJC) updated its diagnostic imaging requirements for hospitals and ambulatory care facilities, with changes effective on July 1, 2015. Among these updated standards are requirements that a diagnostic medical physicist or magnetic resonance imaging (MRI) scientist perform an annual evaluation of image acquisition displays used in computed tomography (CT), MRI, nuclear medicine (NM), and positron emission tomography (PET).^(1)^

An evaluation of “maximum and minimum luminance, luminance uniformity, resolution, and spatial accuracy”^(1)^ are required, although specific testing methods and metrics for evaluating these performance characteristics are not mentioned. The American Association of Physicists in Medicine (AAPM) Online Report No. 3, commonly referred to as TG‐18,^(2)^ provides guidance for the assessment of the performance of displays used in medical imaging, as well as performance standards for both “primary” and “secondary” displays. As defined by TG‐18, a “primary” display refers to one used for interpretation of medical images, such as a radiologist review workstation. A “secondary” display is one that is used for purposes other than medical interpretation. As such, the standards for secondary displays, as defined by TG‐18, define performance criteria for displays used by technologists during image acquisition. In addition to TG‐18, the American College of Radiology (ACR) has provided some performance criteria for acquisition displays in the 2012 Computed Tomography Quality Control Manual^(3)^ (2012 CT QC Manual) and the 2015 Magnetic Resonance Imaging Quality Control Manual^(4)^ (2015 MRI QC Manual). Additionally, acquisition display performance is included in the Accreditation Program Requirements for both ultrasound^(5)^ and nuclear medicine.^(6)^

Many of the performance standards of TG‐18 were defined for cathode ray tube (CRT) displays. Due to the proliferation of liquid crystal display (LCD) technology, AAPM has formed another task group (TG‐270) charged with making recommendations for assessing the quality of flat panel displays. If new performance criteria are to be established, it is first necessary to characterize the performance of existing acquisition displays and compare their performance with existing metrics. The first objective of this work was to evaluate the performance of acquisition displays in clinical use at a large medical center. Measurements included minimum luminance ($L_{\min}$), maximum luminance ($L_{\max}$), luminance uniformity, and a visual evaluation of image quality. The second objective was to compare these observations with existing performance standards and encourage discussion of the appropriateness of these standards.

## II. MATERIALS AND METHODS

Luminance measurements and visual evaluation were performed on (N=42) acquisition displays across multiple imaging modalities including CT (N=5), MRI (N=4), NM and PET (N=8), ultrasound (N=18), and general radiography (N=7). The CT, MRI, NM, PET, and ultrasound (US) displays used in this study represent all of the acquisition displays currently in clinical use within a single facility's Department of Radiology. Among general radiography units, the displays tested included all of the facilities digital radiography systems, with the exception that digital portable units were excluded. All units tested were flat panel displays (no CRTs), and were produced by a variety of manufacturers. The age and amount of use of these displays varied substantially. Some of the displays evaluated had been in use for approximately ten years, while others had less than one year of use. Many of these devices allowed the user to adjust the brightness and contrast. To help standardize the measurement procedure, devices with adjustable levels were set to a brightness of 100% and a contrast of 50%. All luminance measurements were made using a calibrated RaySafe Unfors Xi luminance meter (Billdal, Sweden). No evaluation of the reproducibility of these measurements was performed since previous work demonstrated that this device has a coefficient of variation of less than 1% across a wide range of luminance values ($0.67\;\text{cd}/m^{2}$ to $524.0\;\text{cd}/m^{2}$ It should be noted that for most of these displays, images are not displayed over the entire field of view. Rather, there is a designated location, typically in the center, to display images and the edges are reserved for image acquisition and processing parameters. Consequently, all measurements performed during this study were limited to the field of view where images were displayed, and excluded portions of the display used for other purposes.

## A. Evaluation of minimum and maximum luminance

The 2012 CT ACR Manual recommend using the Society of Motion Picture and Television Engineers (SMPTE) test pattern to evaluate display performance. For this reason, $L_{\min}$ and $L_{\max}$ were measured using either the SMPTE test pattern or the comparable TG‐18 QC pattern, depending on which was available for the acquisition workstation to be tested. For the majority of systems, at least one of these test patterns was available through software installed by the vendor. For systems that did not have test patterns stored locally (N=8), a SMPTE pattern was sent to the system via the facility's Picture Archiving and Communication System (PACS). Single measurements of $L_{\min}$ and $L_{\max}$ were made using the minimum and maximum luminance patches. The meter used in this study is fitted with a flange, provided by the manufacturer, which minimizes the effects of ambient light. It should be noted that measurement using both contact luminance meters which exclude ambient light, and telescopic luminance meters which include ambient light, are supported by TG‐18.

The meter was placed in contact with the display, and contact was maintained until the measured values had stabilized. It should be noted that this device has a 10 mm, circular field of measurement. Consequently, luminance measurements made using the steps of the SMPTE pattern which extend a few centimeters in each direction can be made without including signal from adjacent areas. Care was taken to ensure that pressure on the display was minimal and did not result in alteration in output. After measurement, the mean value, standard deviation (SD), and coefficient of variation were calculated for $L_{\min}$ and $L_{\max}$.

### B. Quantitative evaluation of luminance uniformity

Evaluation of luminance uniformity can be difficult to perform on acquisition workstations as there may be no test pattern readily available that is appropriate for this measurement. For the majority of displays, an open document, image, or window that displays a blank white screen may be used to evaluate uniformity at maximum luminance. To allow luminance uniformity measurements to be made on all displays, a combination of these methods was used. An exception to this was the displays for the ultrasound (US) systems which had a preloaded TG‐18‐UN80 test pattern available that was used for uniformity measurements. For each display, luminance was measured in the center and four corners of the area used to display images. The maximum luminance deviation (MLD) was calculated as (${(L}_{\max}-L_{\min})/(L_{\max}+L_{\min})\times 200$ for each display.^(2)^The mean value, standard deviation, and range were calculated for MLD. Because the luminance of the TG‐18 UN80 pattern is lower than that of a white image, these values were calculated separately for the group of US displays. A *t*‐test was performed to determine if there was a statistically significant difference in the mean MLD values of US and non‐US displays.

### C. Visual evaluation

Both the SMPTE test pattern and the TG‐18 QC test pattern have a number of features that may be employed as part of a visual evaluation of display performance. Many of these features were designed to evaluate CRT displays. For the purpose of this work, visual evaluation was limited to an inspection of the 0%/5% and 95%/100% contrast patches, an evaluation of the spatial resolution patterns, and an evaluation of the grid pattern for distortions. These tests were selected because they can be performed with either the SMPTE or TG‐18 QC test pattern and were a necessary part of evaluating CRT displays. When evaluating the contrast patches and spatial resolution patterns, the visual inspection simply determined if both patches could be seen and all bars of the resolution patterns could be resolved without aliasing. For the grid pattern, distortion was classified as any visible deviation (bowing or bending of the lines) from a rectangular grid. Additionally, a visual evaluation of local non‐uniformities (i.e., dark or bright spots) was performed using either white and black screens or the TG‐18 UN80 and UN10 test patterns.

Visual inspection was performed by one of two observers. All MRI, CT, NM, PET, and general radiography displays were evaluated by a single observer (Observer A); all US displays were evaluated by a second observer (Observer B). While there was no formal analysis of inter‐observer variability, Observer B was trained by Observer A in how to perform a visual evaluation of the test patterns.

## III. RESULTS

### A. Evaluation of minimum and maximum luminance

The mean values for $L_{\min}$ and $L_{\max}$ for the displays tested in this study were $0.28\pm 0.13\;\text{cd}/m^{2}$ and $135.07\pm 33.35\;\text{cd}/m^{2}$, respectively. $L_{\min}$ ranged from 0.09 to $0.63\;\text{cd}/m^{2}$ and $L_{\max}$ ranged from 71.76 to $236.40\;\text{cd}/m^{2}$.

### B. Quantitative evaluation of luminance uniformity

The mean MLD for non‐US displays was 14.47%±5.36% with values ranging from 4.88% to 28.88%. The mean MLD for US displays was 12.61%±4.85% with values ranging from 5.53% to 14.70%. The difference in MLD of US and non‐US displays was statistically significant (p=0.004).

### C. Visual evaluation

Most displays with adjustable brightness and contrast settings, and all displays with fixed settings, were able to distinctly display the 0%/5% and 95%/100% contrast patches. For adjustable displays that were initially set to different brightness and contrast values before testing, two were unable to distinctly display the 5% patch inside the 0% square and one was unable to distinctly display the 95% patch within the 100% square. Adjusting brightness to 100% and contrast to 50% made the 5% patches visible on the displays in question. However, this adjustment did not result in improved visibility of the 95% patch on the display that failed.

For systems that were able to display vendor‐supplied test patterns (N=34), all resolution patterns were resolvable. For the systems where a SMPTE pattern was loaded from PACS (N=8), all displays had aliasing for the smallest resolution pattern. There were no visible distortions of the grid pattern on any of the displays tested. Finally, subtle local nonuniformities were observed on a number of displays, including dark and bright spots and scratches on the surface. Only one display showed a substantial nonuniformity in the form of an uncharacteristically bright spot, approximately 1 cm across, that did not change in luminance regardless of the driving level displayed at that location.

## IV. DISCUSSION

### A. Luminance response and uniformity

For convenience, Table 1 provides a summary of the quantitative results of this study as well as the performance criteria from the ACR QC Manuals. Both the CT (2012) and MRI (2015) QC Manuals require that $L_{\min}$ be no greater than $1.2\;\text{cd}/m^{2}$ and $L_{\max}$ no less than $90\;\text{cd}/m^{2}$ for acquisition displays.^3^, ^4^ While all displays evaluated in this study had an $L_{\min}$ below $1.2\;\text{cd}/m^{2}$, four displays had an $L_{\max}$ below $90\;\text{cd}/m^{2}$. Two of these were acquisition displays for gamma cameras and one was for a PET/CT system. For these three, each system had adjustable brightness and contrast with values set to 100% and 50%, respectively. It should be noted that each of these systems was approaching 10 years of use without replacement of the displays. The other display that had an $L_{\max}$ below $90\;\text{cd}/m^{2}$ was for an MRI system with less than one year of use. Brightness and contrast of this display is not directly adjustable by the user.

**Table 1:** The table provides the mean value, SD, and performance criteria provide by the ACR for $L_{\min}$, $L_{\max}$, and MLD. Additionally, the number of displays that meet each value is included. It should be noted that the performance criterion provided by the ACR for MLD is 15% for CT and 30% for MR

	*Mean*	*SD*	*Performance Criterion*	*Meeting Criterion?*
$L_{\min}(\text{cd}/m^{2})$	0.28	0.13	1.2	42/42
$L_{\max}(\text{cd}/m^{2})$	135.07	33.35	90	38/42
MLD (%)	12.61	4.85	15 (30)	35/42 (42/42)

The ACR CT QC Manual recommends using the SMPTE, or equivalent, test pattern for measurements of $L_{\min}$ and $L_{\max}$. The advantage is that the SMPTE or TG‐18 QC test patterns are often readily available, and multiple evaluations of display performance can be made from a single test pattern. In a busy clinical environment, especially where physics support is provided by consultants rather than in‐house physics support, effective QC testing must also be efficient. However, using a single test pattern to measure $L_{\min}$ and $L_{\max}$ may convolve measurements of minimum and maximum luminance with spatial luminance nonuniformities across the field of the display. This effect may be minimized by using a test pattern where the minimum and maximum brightness patches are located in the center of the image, such as the TG‐18 LN‐12–1 and TG‐18‐LN‐12–18 test patterns. Unfortunately, this increases the complexity of the test procedure, both in the time needed to load the test patterns onto the acquisition systems and to perform the luminance measurements. Further, luminance nonuniformities are typically not spatially linear. In other words, luminance falls off dramatically at the corners or edges of the display rather than gradually across the display. So even in cases where luminance nonuniformity affects measurements of $L_{\min}$ and $L_{\max}$, these effects are likely to be small, given that the 0% and 100% patches of the SMPTE pattern are typically within 30% of the center of the display.

Both the CT QC and MRI QC manuals appear to use TG‐18 as their primary reference.^(3,4)^Regarding $L_{\max}$, TG‐18 suggests that the value of $L_{\max}^{'}$, which is the sum of $L_{\max}$ and the ambient luminance, should exceed $100\;\text{cd}/m^{2}$ for secondary displays.^(2)^ Clearly ambient luminance will vary from system to system and facility to facility. Additionally, luminance meters designed to be used in direct contact with displays are not typically able to evaluate the ambient luminance. Clearly, measurements of $L_{\max}$ will always be lower than measurements of $L_{\max}^{'}$. Consequently, it may have been reasonable for the ACR to set their performance criterion for $L_{\max}$ at a slightly lower value than the TG‐18 criterion for $L_{\max}^{'}$.

The 2012 CT QC Manual requires that MLD should not exceed 30% for CRT displays and 15% for flat panel displays.^(3)^ This echoes the recommendations of TG‐18. The 2015 MRI QC Manual states that MLD must be less than 30% for all displays.^(4)^ Of the displays tested in this study, all had an MLD of less than 30%, but seven displays had an MLD of greater than 15%. It should be noted that the two highest MLD values (approximately 24% and 28%) were for SPECT/CT systems with less than three years of use.

While $L_{\min}$, $L_{\max}$, and MLD are easy to measure, the usefulness of these measurements as a part of routine quality assurance remains a matter of debate. Certainly, an uncharacteristically low $L_{\max}$ can be indicative that a system is not performing as designed. However, what is the clinical impact of a display that has an $L_{\max}$ of $88\;\text{cd}/m^{2}$ or an MLD of 16%, and does this affect the ability of the technologist to effectively and safely operate the modality? To address this issue it may be important to consider the individual tasks for which these displays are being used. TG‐18 suggests that displays used in medicine should comply with the DICOM Standard Grayscale Display Function,^(2)^ but this is probably more important for acquisition workstations where the technologist manipulates image data and performs postprocessing than for displays simply used to determine if the patient was properly positioned. For displays where no image manipulation is performed and the technologist is not attempting to match what they see with what will be displayed to the radiologist, a visual evaluation of display performance may be sufficient.

### B. Luminance uniformity testing methodology

As stated above, the majority of displays evaluated in this study utilized a white screen for the measurements used to calculate MLD. This method has been employed in previous studies seeking to characterize luminance uniformity for primary interpretation displays.^7^, ^8^ The 2012 CT QC manual states that measurements should be made at the center of the display and near all four corners or the center, corners, and all four sides depending on the test pattern used, but does not specifically name any test pattern for this purpose or discuss an appropriate driving level.^(3)^ The 2015 MRI QC manual states the measurements should be made at the center and four corners for both the white screen ($L_{\max}$) and a dark screen ($L_{\min}$).^(4)^ The TG‐18 report suggests using both the TG‐18‐UN10 and TG‐18‐UN80 test patterns for luminance uniformity. As previously mentioned, the luminance uniformity performance criteria utilized by the ACR appear to have come from TG‐18. Whether or not luminance uniformity calculated at $L_{\max}$ should meet a standard established for a lower luminance remains to be determined. A limitation of this study is that US displays that were tested using the TG‐18‐UN80 test pattern were not also evaluated at maximum luminance to determine if significant changes in luminance uniformity are observed as brightness increases. While the mean MLD for the US displays was found to be statistically significantly different than for the others evaluated, it is not clear whether this is the result of the test pattern used or the display models tested.

### C. Display contrast

Due to the important role that luminance plays in contrast visibility,^(9)^ display quality assurance often includes characterization of luminance response across a range of driving levels and comparison to the DICOM GSDF curve. While this is standard practice for displays used for primary interpretation, it is often impractical to implement for acquisition displays. Additionally, while analysis of the GSDF curve is excellent for ensuring consistency between monitors, it has limitations in characterizing display contrast. The GSDF curve is based on contrast visibility studies performed under variable adaptation conditions, but viewing of diagnostic images occurs under fixed adaptation conditions. Under fixed adaptation conditions, the visual contrast response is worst for the brightest and darkest parts of a diagnostic image.^(10)^ Consequently, deficiencies in display contrast will be most apparent at the extreme ends of the display gray scale. This supports the adequacy of a visual assessment of the 0%/5% and 95%/100% contrast patches for evaluating display contrast.

As previously mentioned, deficiencies in display contrast for acquisition displays were observed as part of this study, but often can be corrected by adjusting the display brightness and contrast settings. The only display in this study that was unable to distinctly display the 0%/5% and 95%/100% contrast patches was a newer display, suggesting that poor contrast performance is not necessarily age‐related and may warrant vendor intervention.

### D. Spatial resolution and image distortion

As stated in the Materials & Methods section C, when a SMPTE test pattern was sent to any unit from PACS (as opposed to using a vendor‐supplied test pattern), aliasing of the smallest resolution patterns was observed for all displays. It should be noted that, over the years, there have been multiple iterations of the SMPTE test pattern with variations in the line spacing of the resolution patterns. As a test, a different version of the SMPTE pattern with a wider line spacing for the smallest resolution pattern was sent to the systems where aliasing was observed. When this pattern was displayed, no aliasing was observed. This finding highlights two important points. First, the results of a visual evaluation of display performance are directly dependent on the appropriate choice of test pattern, even among different versions of the SMPTE pattern, which are often assumed to be identical. Second, vendors are unlikely to install test patterns that will result in their displays failing visual inspection. This highlights the importance of knowing which version of the SMPTE pattern is being used for display evaluation, and encourages consistent use of a specific version.

Ultimately, this brings up the question of whether a visual evaluation of resolution for flat panel displays is useful. The level of detail that these displays can resolve is primarily governed by the pixel pitch^(11)^ and is unlikely to vary during the lifetime of the display. Additionally, no display in this study showed visible signs of distortion. While pincushion, barrel, and skew distortions were relatively common for CRT displays,^(2)^ the mechanisms by which they occur are not a concern for flat panel displays. The ACR has included both resolution and distortion tests as part of routine quality assurance for both CT and MRI systems, but it appears that these evaluations are irrelevant in assessing performance of flat panel displays.

### E. Local nonuniformities

There were several small area nonuniformities found in this study, including bright and dark spots and scratches. There was one instance of a large (∼1 cm diameter) nonuniformity. It has been demonstrated that even a single defective pixel influences visual perception of a large area around the affected pixel^(12)^ and some have shown that local nonuniformities negatively affect visual search performance.^(13)^ While this is an important performance aspect of primary displays, it is questionable whether the effect is relevant for acquisition displays.

### F. Additional limitations

One objective of this work was to capture a “snapshot” of the performance of the acquisition displays in clinical use at a large medical center. As such, these data provide a general sense of how acquisition workstations perform and what to expect when evaluating them. However, this study is subject to several limitations that should be considered when interpreting the results. First, it should be noted that while a total of 42 displays were evaluated, the number of displays in each modality was limited to single digits, with the exception of ultrasound. Attempts to characterize the performance of displays for any particular modality would require a wider selection of manufacturers and models, and a greater total number of displays than was available. Among ultrasound displays evaluated, 16 of 18 were the same model. In this case, some claims regarding the performance of that particular model would be possible, but the data cannot be extrapolated to apply to all US systems. Another limitation to this study is that none of the data were directly correlated with display use. Ideally, a comparison would be made between $L_{\max}$ and MLD and the number of backlight hours to determine if there is a correlation these performance metrics and display usage. Unfortunately, determining the number of backlight hours of an acquisition display can be difficult or impossible, and therefore limits estimates of usage to a simple tracking of installation date.

## V. CONCLUSIONS

There are an increasing number of accreditation organizations that require testing of medical imaging acquisition displays, although specific performance criteria are rarely provided. There is currently very little information about the performance characteristics of acquisition displays used in a clinical setting, and what performance criteria should be used during testing. This work helps fill that gap by quantitatively evaluating minimum luminance, maximum luminance, and luminance uniformity, as well as visually evaluating contrast, spatial resolution, distortion, and local nonuniformities for 42 acquisition displays across several modalities. The results were compared with existing performance standards, provided by ACR and AAPM.

An effective quality control program needs to include tests and acceptable performance criteria that are both meaningful and relevant to the equipment's intended use. Acquisition displays have a variety of potential roles in the clinic. Some are used by the technologist for a gross evaluation of image quality. In such cases, the user may only need to be able to tell if the correct anatomy was scanned, view overall anatomy to aid in patient positioning, or tell if there was excessive patient movement during the exam. A more detailed display analysis may not be necessary if the user is not looking for small image details. In some settings, however, the user may need to perform postacquisition image processing before sending the images to PACS. In this case, the user may need to ensure that the image appearance on the acquisition display is the same as on the primary display used for image interpretation. In the case of ultrasound, the user may rely heavily on the image appearance on the acquisition displays in order to make clinical decisions, making the role of the acquisition display very similar to that of the primary display. Clearly, the performance requirements are different in each of these settings, highlighting the importance of considering what the performance goals are for a specific piece of equipment and of designing a quality control program to meet those needs.

## COPYRIGHT

This work is licensed under a Creative Commons Attribution 3.0 Unported License.
